# Hirschsprung-associated enterocolitis (HAEC): a significant complication of Hirschsprung’s disease that may benefit from decompressive endoscopy

**DOI:** 10.1093/gastro/goag113

**Published:** 2026-11-10

**Authors:** Óscar Moralejo Lozano, Natalia María Arietti López, Vanessa Camila Jarrín Pesantes, Rafael Ángel Gómez Rodríguez

**Affiliations:** Gastroenterology and Hepatology Department, Complejo Hospitalario Universitario de Toledo, Toledo, Spain; Gastroenterology and Hepatology Department, Complejo Hospitalario Universitario de Toledo, Toledo, Spain; Gastroenterology and Hepatology Department, Complejo Hospitalario Universitario de Toledo, Toledo, Spain; Gastroenterology and Hepatology Department, Complejo Hospitalario Universitario de Toledo, Toledo, Spain

## Introduction

Hirschsprung’s disease (HD) is a congenital condition causing intestinal obstruction due to the absence of ganglion cells in the distal bowel. Although surgery often restores function, some patients develop long-term complications such as chronic constipation or Hirschsprung-associated enterocolitis (HAEC). HAEC is a serious, potentially fatal condition that can occur both preoperatively and days to years postoperatively. We report a case of a 23-year-old male with prior HD surgery who developed late-onset HAEC. This case underscores the need for clinical awareness and shows that conservative management, including endoscopic decompression, may be effective.

## Case report

We present the case of a 23-year-old male with a history of HD surgically treated at 8 months of age (with histological confirmation that the proximal end of the resected segment presented normal neuronal elements). Since then, the patient has suffered from mild chronic constipation. In August 2024, the patient presents at the Emergency Room with acute generalized intermittent abdominal pain, vomiting, and no bowel movements for the last 48 hours. On physical examination, the patient presents with normal blood pressure but tachycardia, and a distended, tympanic abdomen, with generalized tenderness on palpation. Blood tests show metabolic acidosis with hyperlactatemia, leukocytosis (31 200 neutrophils·10^6^/L [normal range <11 000 neutrophils·10^6^/L]) and C reactive protein of 425.7 mg/L [normal range <5 mg/L].

An emergent CT scan revealed a pronounced pancolonic dilation, with abundant faecal content, and pneumatosis in the distal colon (Fig. 1A and B). The patient is diagnosed with HAEC grade 3 and admitted to the ICU and started on conservative treatment including crystalloid rehydration, vasopressor support, broad-spectrum antibiotic therapy (ceftriaxone and metronidazole, 7 days), laxatives (polyethylene glycol 3350, 13.8 g, three times a day), and cleansing enemas. A decompressive endoscopy is performed within the first hours. Colonoscopy is performed reaching a 50 cm distance from the anal verge. From the upper rectum, a black mucosa is present, without ulceration or bleeding (Fig. 1C). On withdrawal, as much aspiration as possible is performed to reduce distention of the intestinal wall, trying to improve intestinal perfusion by decreasing luminal pressure. Immediate clinical improvement occurs. The patient recovered and was discharged 11 days after the episode, asymptomatic, with chronic laxative treatment. There were no further episodes in the following 9 months.

**Figure 1:**
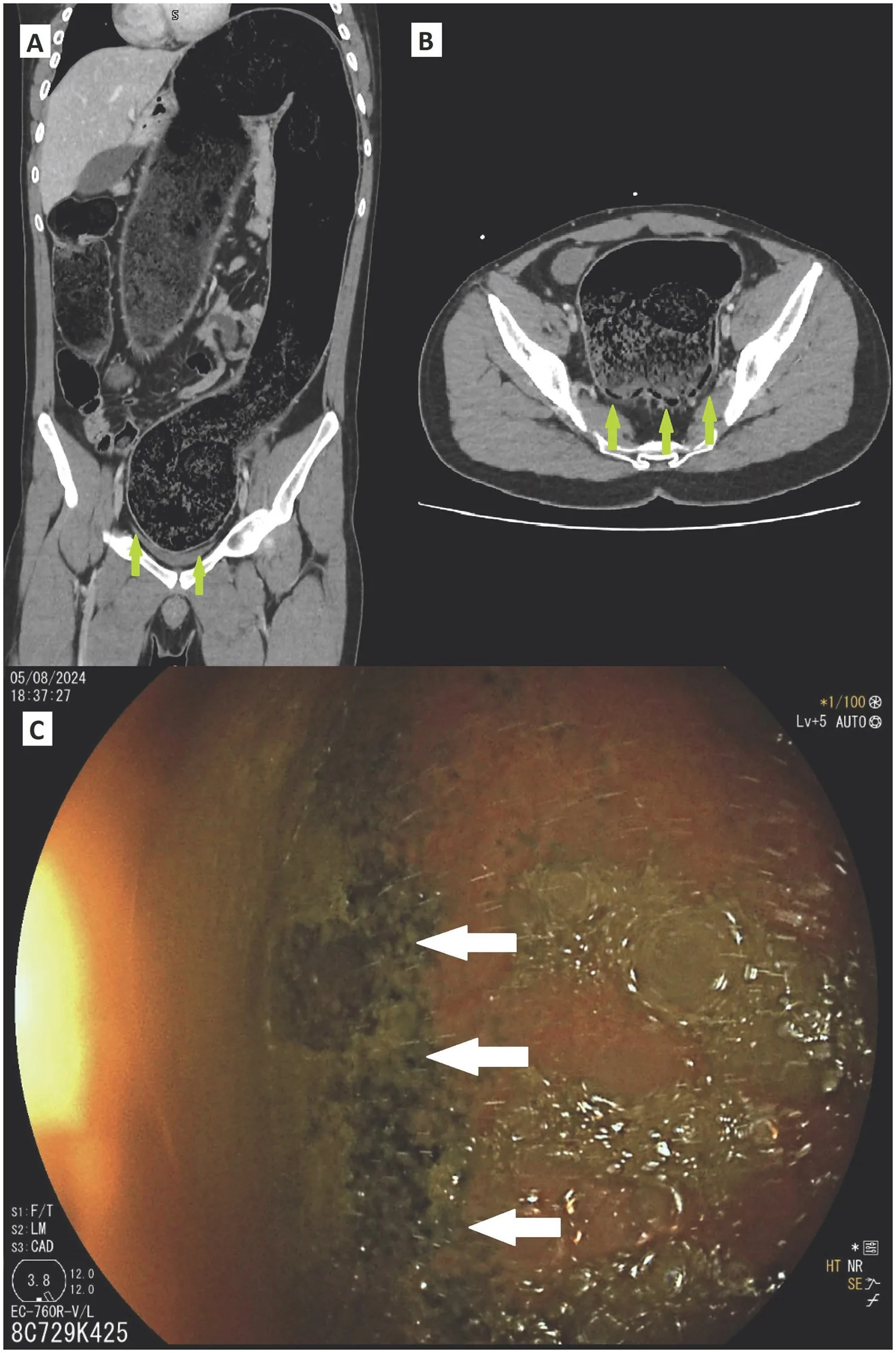
(A) and (B): CT scan with colonic dilation and faecal content. Green arrows point to pneumatosis in the wall of the rectum, a sign of ischaemic mucosa. (C) Endoscopic view of the upper rectum where a black plaque in the mucosa is visible, indicating ischaemic mucosa.

## Discussion

HD is a congenital disorder causing bowel obstruction and the surgical correction follows confirmed aganglionosis [1, 2]. While most patients experience normal bowel function after surgery, a subset may continue to suffer from abdominal distension, chronic constipation, and HAEC [3]. HAEC can occur both pre- and postoperatively. Postoperative HAEC occurs in 25%–37% of cases, and ∼65% occur during the first year after surgery. It carries an average death rate ranging from 1% to 10% [4]. It can happen days or up to >50 years after surgery. The pathogenesis of HAEC remains incompletely understood, but proposed mechanisms include bacterial overgrowth, impaired mucosal immunity, abnormal colonic motility, and persistent dysbiosis [5]. Histological factors such as residual aganglionic segments, transition zone pull-through, or acquired hypoganglionosis may predispose patients to HAEC [1, 6, 7]. Mechanical issues such as anastomotic strictures, bowel torsion, or motility disorders from scarred or denervated bowel may also contribute [1].

Clinical presentation ranges from mild gastrointestinal symptoms to fulminant sepsis. Diagnostic criteria and severity grading systems are useful to guide treatment [8]. HAEC is graded as: Grade 1 with mild gastrointestinal symptoms and minimal distension; Grade 2 with systemic illness, explosive diarrhoea, abdominal distension/tenderness, and radiographic ileus; and Grade 3 (severe) with shock or peritonitis, marked distension, and pneumatosis or pneumoperitoneum. In Grade 3 cases, surgery may be needed, though conservative management can be attempted initially in appropriate candidates.

Decompressive endoscopy is not currently mentioned in clinical practice guidelines for the management of HAEC [8]. Decompressive colonoscopy, however, has been successfully applied in other scenarios involving colonic distension, such as sigmoid volvulus and acute colonic pseudo-obstruction. The procedure has not been assessed in randomized trials, but there are observational reports on acute colonic pseudo-obstruction [9, 10]. Decompressive colonoscopy should be performed with minimal air insufflation, preferably carbon dioxide rather than air. No oral cathartic preparation should be used, but a cleansing enema before the procedure should be considered. The colonoscope should be advanced as far as possible, although careful manipulation is essential to avoid iatrogenic injury, particularly colonic perforation. As a result, in most cases, only the left colon or distal transverse colon is reached. During withdrawal, extensive air suctioning must be performed. In the setting of acute colonic pseudo-obstruction, a perforation rate of <1% has been reported [10].

Whether decompressive colonoscopy is beneficial in HAEC remains to be determined. It may help reduce intraluminal pressure and consequently improve intestinal wall perfusion, potentially preventing bowel perforation due to transmural ischaemic necrosis. Further studies are needed to evaluate the role and safety of decompressive colonoscopy in this clinical setting.
